# Loss of PARP-1 attenuates diabetic arteriosclerotic calcification via Stat1/Runx2 axis

**DOI:** 10.1038/s41419-019-2215-8

**Published:** 2020-01-10

**Authors:** Peng Li, Ying Wang, Xue Liu, Bin Liu, Zhao-yang Wang, Fei Xie, Wen Qiao, Er-shun Liang, Qing-hua Lu, Ming-xiang Zhang

**Affiliations:** 1 Department of Pharmacology, College of Pharmacy, Xinxiang Medical University, Xinxiang, China; 2 The Key Laboratory of Cardiovascular Remodeling and Function Research, Chinese Ministry of Education, Chinese Ministry of Health and Chinese Academy of Medical Sciences, Department of Cardiology, Qilu Hospital of Shandong University, Jinan, China; 3 Department of Cardiology, The Second Hospital of Shandong University, Jinan, Shandong China

**Keywords:** Differentiation, Calcification

## Abstract

Accelerated atherosclerotic calcification is responsible for plaque burden, especially in diabetes. The regulatory mechanism for atherosclerotic calcification in diabetes is poorly characterized. Here we show that deletion of PARP-1, a main enzyme in diverse metabolic complications, attenuates diabetic atherosclerotic calcification and decreases vessel stiffening in mice through Runx2 suppression. Specifically, PARP-1 deficiency reduces diabetic arteriosclerotic calcification by regulating Stat1-mediated synthetic phenotype switching of vascular smooth muscle cells and macrophage polarization. Meanwhile, both vascular smooth muscle cells and macrophages manifested osteogenic differentiation in osteogenic media, which was attenuated by PARP-1/Stat1 inhibition. Notably, Stat1 acts as a positive transcription factor by directly binding to the promoter of Runx2 and promoting atherosclerotic calcification in diabetes. Our results identify a new function of PARP-1, in which metabolism disturbance-related stimuli activate the Runx2 expression mediated by Stat1 transcription to facilitate diabetic arteriosclerotic calcification. PARP-1 inhibition may therefore represent a useful therapy for this challenging complication.

## Introduction

Vascular calcification is prevalent in patients suffering from diabetes and has been strongly associated with adverse cardiovascular outcome^1,2^. In particular, under procalcific stimuli common in diabetes such as inflammation, oxidative damage, high glucose (HG), and inorganic phosphate, several types of vascular cells including vascular smooth muscle cells (VSMCs) and macrophages can undergo a phenotypic switch to osteoblast-like cells^3–5^. In turn, arteriosclerotic calcification, which represents a significant predictor of susceptibility to plaque rupture, is directly driven by the osteogenic differentiation of VSMCs and the shift of macrophage phenotypes^6,7^. Furthermore, although some in vitro studies have shown that high concentrations of glucose, a characteristic feature of diabetes, can accelerate osteogenic differentiation and calcification^8^, the underlying molecular mechanisms are still poorly understood and it is not clear to what extent hyperglycemia contributes to the process of arteriosclerotic calcification in vivo.

The Runt-related transcription factor (Runx2/Cbfa1) plays a central role in osteoblast differentiation^6^. Runx2 binds to the osteoblast-specific cis-acting element 2, which is found in the promoter regions of both osteocalcin and alkaline phosphatase^9^, and thus regulates their expression. This may explain the requirement for Runx2 as a molecular switch for osteogenic differentiation and the inability of any parallel pathway to overcome its absence. The expression of Runx2 is low in normal vascular cells but becomes elevated in atherosclerotic calcified lesions from human and mouse specimens^6,10,11^. Furthermore, studies have shown that diabetes leads to the misregulation of Runx2 expression and aggravates aortic stiffening in vivo and in vitro^12^. Conversely, the targeted disruption of Runx2 appears to be beneficial for attenuating atherosclerotic calcification^6,13^, especially in diabetes.

Poly [ADP-ribose] polymerase 1 (PARP-1), the most abundant isoform of PARP^14^, participates in multiple pathophysiological processes including DNA repair, gene transcription, and cell death by targeting genes such as AP-1^15–17^. Several recent studies have reported that PARP-1 could negatively regulate bone resorption and that PARP inhibition could suppress the osteogenic differentiation of osteosarcoma cells and mouse mesenchymal stem cells^18,19^. We have shown that PARP-1 functions as a crucial modulator of NF-kB activity and that PARP-1 inhibition attenuates inflammatory responses during atherogenesis^20,21^ and diabetic complications^22^. However, the potential link between PARP-1 regulation and diabetes-induced atherosclerotic calcification has not been examined. We therefore speculated that PARP-1 could modulate osteogenic differentiation and thus contribute to atherosclerotic calcification in diabetes.

In the present study, we first show that HG directly drives atherosclerotic calcification by promoting Runx2 expression in both VSMCs and macrophages. In addition, PARP-1 inhibition downregulates Stat1/Runx2 transcriptional activity and alleviates atherosclerotic calcification in diabetes. We further demonstrate that Stat1 directly binds to the Runx2 promoter and regulates its transcription. Accordingly, Stat1 overexpression in vitro aggravates osteogenic differentiation via the upregulation of Runx2. Our results thus shed new light on the potential contribution of PARP-1 in the regulation of osteogenic differentiation of both VSMCs and macrophages.

## Results

### PARP-1 inhibition attenuates atherosclerotic calcification and decreases vessel stiffening in ApoE^−/−^ mice and diabetic ApoE^−/−^ mice

**Fig. 1:**
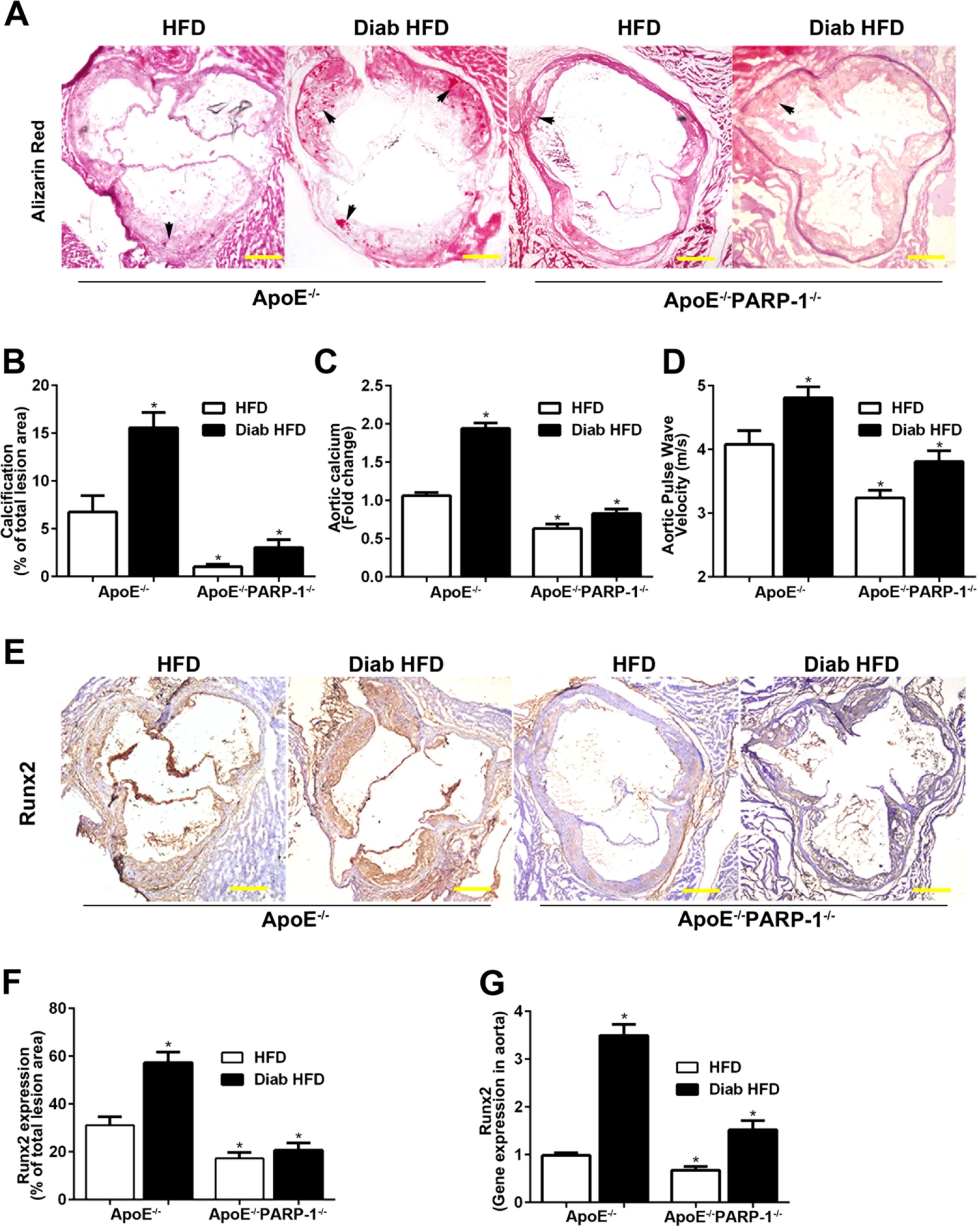
PARP-1 deletion attenuates diabetic atherosclerotic calcification. **a**, **b** PARP-1 deletion decreased Alizarin Red positively stained atherosclerotic lesion area in diabetic ApoE^−/−^ mice. **c**, **d** PARP-1 deletion decreased aortic calcium content and pulse wave velocity. Arrows indicate calcium mineral deposition. **e**, **f** Immunohistochemical staining shows increased Runx2-positive areas in diabetic ApoE^−/−^ mice. **g** Real-time PCR shows increased Runx2 gene expression in the aortas of diabetic ApoE^−/−^ mice. Scale bar = 100 µm. Bar values represent the means ± SD. *n* = 8 in each group. Asterisks indicate statistically significant differences (*P < 0.01, vs. ApoE^−/−^ HFD mice). The statistical tests are justified as appropriate and meet the assumptions of the tests. The variance between the groups is similar.

PARP-1 deletion inhibited atherosclerotic calcification, reduced the content of aortic calcium, and decreased vessel stiffening in ApoE^−/−^ mice challenged with High-Fat Diet (HFD) for 12 weeks (Fig. 1a–d). However, these findings were more obvious in diabetic ApoE^−/−^ mice. Calcium nodules were significantly increased in the diabetic atherosclerotic lesion areas (15.83 ± 1.46% vs. 6.80 ± 1.46%, *p* < 0.01), as determined by Alizarin Red staining (Fig. 1a, b). In accordance with the content of aortic calcium, aortic stiffness was increased in diabetic ApoE^−/−^ mice as determined by pulse wave velocity (PWV) using echocardiography (Fig. 1c, d). As compared with diabetic ApoE^−/−^ mice, PARP-1 deletion significantly attenuated diabetic atherosclerotic calcification (3.14 ± 0.78% vs. 15.83 ± 1.46%, *p* < 0.01, Fig. 1b), reduced the content of aortic calcium (0.84 ± 0.06 vs. 1.95 ± 0.07 fold-change, *p* < 0.01, Fig. 1c), and decreased vessel stiffening (3.80 ± 0.15 vs. 4.84 ± 0.17, *p* < 0.01, Fig. 1d). After 12 weeks of diabetes, diabetic mice showed higher levels of blood glucose, total plasma cholesterol, and triglyceride, whereas PARP-1 deletion had no obvious effect on mice body weight, blood glucose, total cholesterol, triglyceride, serum calcium, or phosphorus levels (Table S1).

To gain further insight into the effect of PARP-1 on diabetic atherosclerotic calcification, we detected the expression of Runx2 in the aortas as well as atherosclerotic lesions. Immunohistochemical staining showed increased Runx2-positive areas in diabetic atherosclerotic lesion (Fig. 1e, f). Furthermore, Real-time PCR showed increased Runx2 gene expression in the aortas of diabetic ApoE^−/−^ mice, which was reversed by PARP-1 deletion (Fig. 1g). These data indicated that PARP-1 deletion may attenuate diabetic atherosclerotic calcification by targeting Runx2.

To investigate whether PARP inhibitor PJ34 treatment could prevent diabetic arteriosclerotic calcification, diabetic ApoE^−/−^ mice on HFD were injected intraperitoneally with PJ34 at a dose of 10 mg/kg every other day for 12 weeks. As expected, PJ34 treatment attenuated atherosclerotic calcification (Supplementary Fig. 1A, B), reduced the content of aortic calcium (Supplementary Fig. 1C), and decreased vessel stiffening (Supplementary Fig. 1D). Moreover, we also found that the PARP inhibitor PJ34 prevented VSMCs calcification and Raw264.7 macrophages calcification in vitro (Supplementary Fig. 2).

### PARP-1 depletion attenuates diabetic atherosclerotic calcification by inhibiting VSMCs calcification and synthetic phenotype switching

**Fig. 2:**
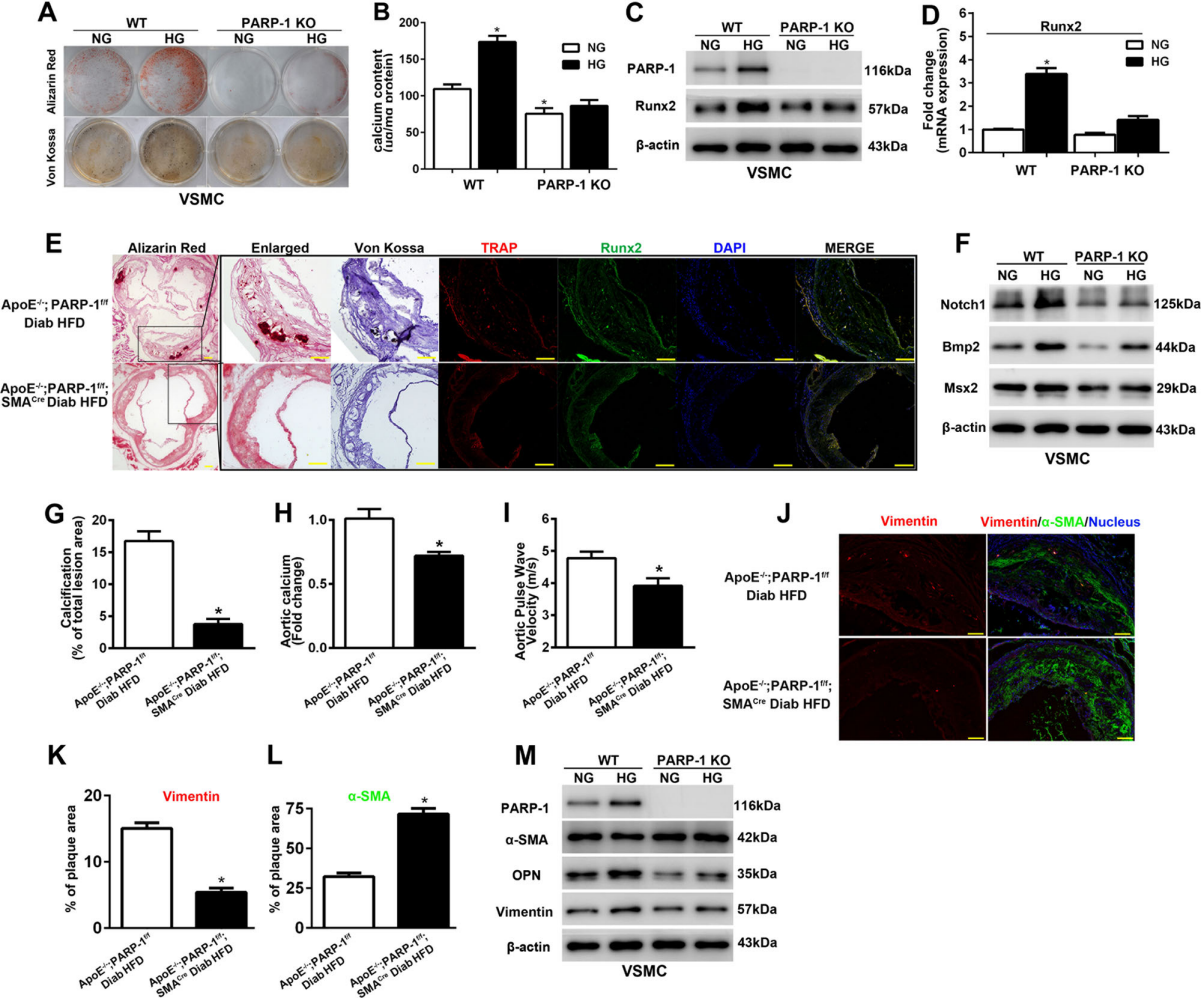
PARP-1 depletion attenuates hyperglycemia-induced VSMCs calcification and synthetic phenotype switching in vitro and in vivo. **a** VSMCs were isolated from WT and PARP-1^−/−^ mice and cultured in osteogenic medium with (HG) or without (NG) high glucose (27.5 mM) for 3 weeks. Calcification was determined by Alizarin red and Von Kossa staining (*n* = 5). **b** Total calcium content was quantified using a Calcium Colorimetric Assay Kit. Results shown are normalized to the total protein amount (*n* = 5). *P < 0.01, vs. WT VSMCs in osteogenic medium without high glucose. **c**, **d** Western blot and RT-PCR analyses were performed to determine the effect of PARP-1 deletion on the expression of Runx2 (*n* = 5). *P < 0.01, vs. WT VSMCs in osteogenic medium without high glucose. **e** Consecutive sections stained with Alizarin Red or Von Kossa to detect calcification, and immunofluorescence staining of TRAP and Runx2 are shown. VSMC-specific PARP-1 deletion inhibited the expression of Runx2 and formation of TRAP-positive cells. Scale bar = 50 µm. **f** PARP-1 depletion attenuates HG-induced VSMCs calcification involving Notch1 repression. Western blot analysis of Notch1 and osteogenic genes including Bmp2, Msx2, and OPN in VSMCs isolated from WT and PARP-1^−/−^ mice (*n* = 5). **g**–**i** VSMC-specific PARP-1 depletion decreased atherosclerotic calcification lesion area, aortic calcium content, and pulse wave velocity (*n* = 8). *P < 0.01, vs. diabetic ApoE^−/−^/PARP-1^f/f^ mice. **j** Immunofluorescence staining of vimentin (red) and α-SMA (green) in atherosclerotic plaques. VSMC-specific PARP-1 depletion reverses hyperglycemia-induced synthetic phenotype switching of VSMCs in atherosclerotic plaques. **k**, **l** Quantification of vimentin and α-SMA coverage. Scale bar = 50 µm (*n* = 8). *P < 0.01, vs. diabetic ApoE^−/−^/PARP-1^f/f^ mice. **m** Western blot analysis of contractile (α-SMA) and synthetic proteins (vimentin and OPN) in VSMCs isolated from WT and PARP-1^−/−^ mice (*n* = 5). The statistical tests are justified as appropriate and meet the assumptions of the tests. The variance between the groups is similar.

To determine the effect of PARP-1 on VSMC calcification, we isolated VSMCs from wild-type (WT) and PARP-1^−/−^ mice. As determined by Alizarin Red and Von Kossa staining, evident calcification was found in VSMCs, especially in those exposed to osteogenic medium with HG. In sharp contrast, PARP-1 deletion attenuated VSMC calcification and decreased calcium content in VSMCs (Fig. 2a, b). These findings were more obvious in HG-cultured VSMCs. In parallel, HG-promoted total calcium content in VSMCs was blocked by PARP-1 deletion (86.45 ± 8.03 vs. 173.83 ± 8.32, *p* < 0.01, Fig. 2b). Furthermore, PARP-1 deletion impeded the HG-induced expression of Runx2 (Fig. 2c, d).

To better understand the role of SMC-specific PARP-1 in the biology of diabetic arteriosclerotic calcification, we generated STZ-induced diabetic ApoE^−/−^/PARP-1 floxed (PARP-1^f/f^) mice and ApoE^−/−^/PARP-1^f/f^/SMA^Cre^ mice. SMC-specific PARP-1 depletion had no obvious effect on the general metabolic parameters of diabetic ApoE^−/−^ mice (Table S2). Conversely, PARP-1 deletion in VSMCs reduced the formation of calcium nodule in the atherosclerotic lesion areas (3.99 ± 1.11% vs. 16.43 ± 1.26%, *p* < 0.01) and exhibited significantly decreased calcium content (0.71 ± 0.02 vs. 1.00 ± 0.05 fold-change, *p* < 0.01) in aortas in comparison with diabetic ApoE^−/−^ mice (Fig. 2e–h). Aortic stiffness was decreased in diabetic ApoE^−/−^/PARP-1^f/f^/SMA^Cre^ mice (3.91 ± 0.16 vs. 4.83 ± 0.15, *p* < 0.01) as determined by echocardiography (Fig. 2i). Hyperglycemia enhanced the formation of tartrate-resistant acid phosphatase (TRAP)-positive osteoclast-like cells, which was coupled with the development of atherosclerotic calcification. PARP-1 deletion inhibited the expression of Runx2 and formation of TRAP-positive cells (Fig. 2e). We further found PARP-1 depletion suppressed the expression of osteogenic genes including Bmp2 and Msx2 induced by HG (Fig. 2f). These data indicated that PARP-1 deletion attenuated hyperglycemia-induced calcification in vitro and in vivo.

As VSMC phenotypic transition has been associated with vascular calcification^23^, we assessed whether PARP-1 deletion affected VSMC phenotypic transition during diabetic atherosclerotic calcification. As illustrated by immunofluorescence staining, the expression of vimentin, a marker of synthetic VSMCs, was significantly decreased in the PARP-1 deletion mice compared with the diabetic ApoE^−/−^ controls (5.36 ± 0.58% vs. 15.21 ± 0.80%, *p* < 0.01), whereas α-SMA was significantly increased in the PARP-1 deletion mice (71.31 ± 3.09% vs. 32.64 ± 2.09%, *p* < 0.01) (Fig. 4j–l). Western blot analysis showed that HG treatment significantly increased the expression of PARP-1 and markers of synthetic VSMCs (vimentin and OPN), but decreased α-SMA expression (Fig. 4m). The results revealed that PARP-1 depletion reversed the process of HG-mediated synthetic phenotypic transition in VSMCs.

### PARP-1 depletion attenuates diabetic atherosclerotic calcification by inhibiting macrophage calcification and macrophage polarization

**Fig. 3:**
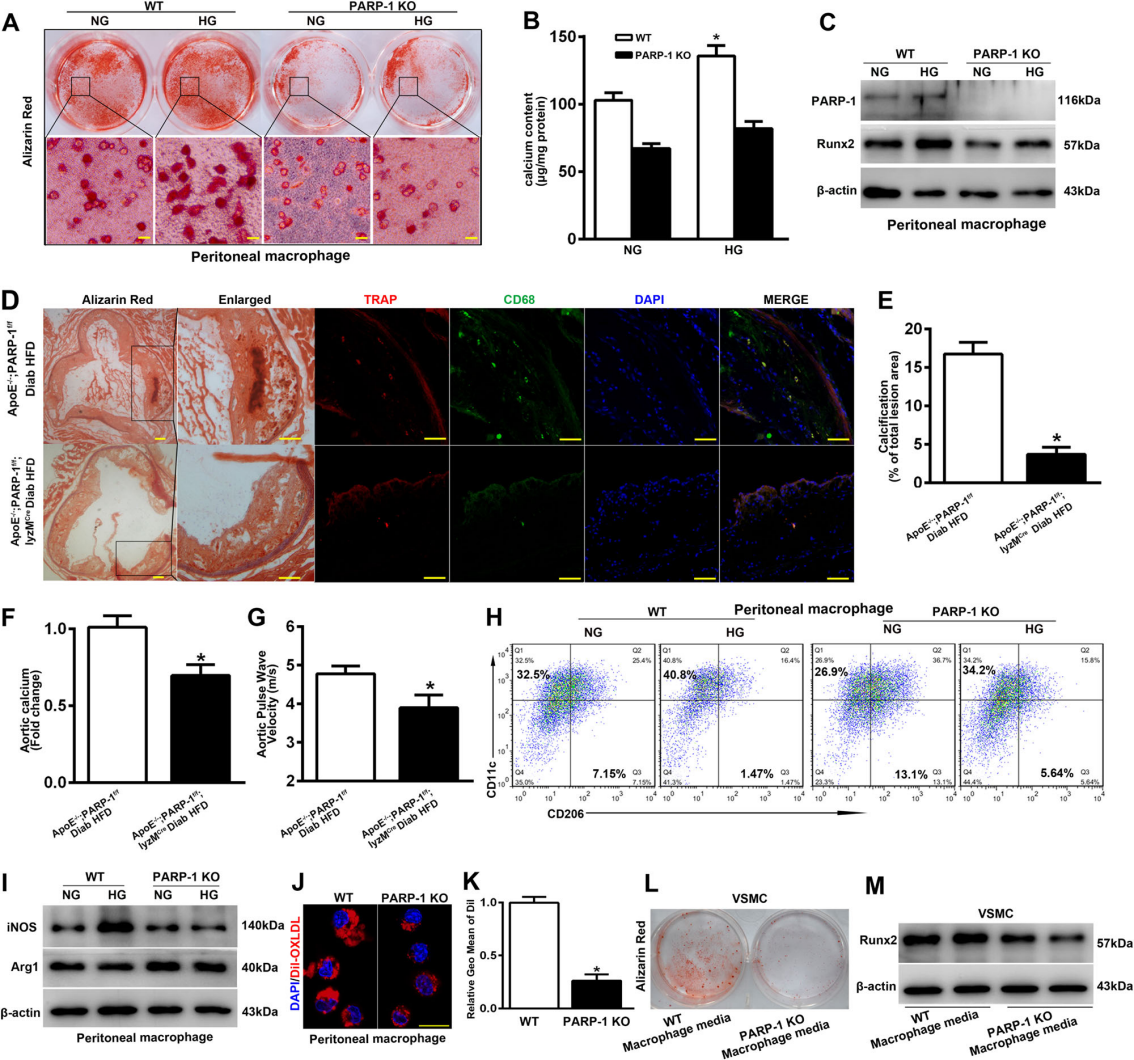
PARP-1 plays a critical role in hyperglycemia-induced arteriosclerotic calcification by regulating macrophage calcification and macrophage polarization. Peritoneal macrophages were isolated from thioglycollate-injected WT and PARP-1^−/−^ mice. **a**, **b** Macrophages exhibited evident calcification and increased calcium content after 3-week exposure to osteogenic medium with high glucose (HG) compared with normal glucose (NG), which was reversed by PARP-1 deletion (*n* = 5). Scale bar = 10 µm. **c** Western blot analysis was performed to determine the effect of PARP-1 deletion on the expression of Runx2 (*n* = 5). **d** Colocalization of TRAP and CD68 revealed that macrophages participated in atherosclerotic calcification. Scale bar = 50 µm. **e**–**g** Macrophage-specific PARP-1 depletion decreased atherosclerotic calcification lesion area, aortic calcium content, and pulse wave velocity (*n* = 8). *P < 0.01, vs. diabetic ApoE^−/−^/PARP-1^f/f^ mice. **h**, **i** Flow cytometry and immunoblotting were performed to detect the effect of PARP-1 depletion on macrophage phenotypes switching. **j**, **k** Effect on endocytosis of Dil-labeled ox-LDL by PARP-1 depletion. Scale bar = 10 µm. *n* = 5. Asterisks indicate statistically significant differences (*P < 0.01). **l**, **m** VSMCs exhibited evident calcification by stimulation with conditioned medium from WT mouse macrophages for 2 weeks, accompanied by higher expression levels of Runx2. PARP-1 depletion abolished the procalcification effect of macrophages (*n* = 5). The statistical tests are justified as appropriate and meet the assumptions of the tests. The variance between the groups is similar.

Given that the contribution of macrophages to atherosclerotic calcification remains controversial, we first investigated whether macrophages could undergo osteogenic differentiation in vitro. Peritoneal macrophages were isolated from thioglycollate-injected WT and PARP-1^−/−^ mice. Both peritoneal macrophages and RAW264.7 macrophages exhibited evident calcification after 3-week exposure to osteogenic medium especially in HG-osteogenic medium cultured macrophages (Fig. 3a, b; Supplementary Fig. 2), whereas PARP inhibition reversed HG-induced macrophage calcification and Runx2 expression (Fig. 3a–c, Supplementary Fig. 2).

To discern the functional impact of PARP-1 depletion in macrophages on diabetic atherosclerotic calcification, we generated diabetic ApoE^−/−^/PARP-1^f/f^/lyzM^Cre^ and ApoE^−/−^/PARP-1^f/f^ mice. Macrophage-specific PARP-1 depletion had no obvious effect on the general metabolic parameters of diabetic ApoE^−/−^ mice (Table S2). Colocalization of TRAP (an osteoclast-like cell marker) and CD68 (a macrophage marker) in calcified atherosclerotic lesions was confirmed by immunofluorescence and revealed that macrophages independently participated in atherosclerotic calcification in vivo (Fig. 3d). In accordance with SMC-specific PARP-1 depletion, PARP-1 deletion in macrophages also reduced the formation of calcium nodules in the atherosclerotic lesion areas (4.06 ± 0.91 vs. 16.43 ± 1.26, *p* < 0.01) and significantly decreased calcium content in the aortas (0.70 ± 0.05 vs. 1.00 ± 0.05 fold-change, *p* < 0.01) in comparison with diabetic ApoE^−/−^ mice (Fig. 3d–f). In addition, aortic stiffness was decreased in diabetic ApoE^−/−^/PARP-1^f/f^/lyzM^Cre^ mice (3.88 ± 0.21 vs. 4.83 ± 0.15, *p* < 0.01) as determined by echocardiography (Fig. 3g). Our results demonstrate the key role of macrophage-specific PARP-1 in the development of arteriosclerotic calcification in diabetes.

To evaluate the macrophage phenotypes in osteogenic differentiation, we used flow cytometry and western blot to analyze cultured peritoneal macrophages. CD11c and iNOS were regarded as M1 macrophages markers, whereas CD206 and arginase 1 (Arg1) were regarded as M2 macrophage markers. Macrophages from WT mice displayed enhanced expression of CD11c and iNOS under HG treatment, whereas the anti-inflammatory phenotype markers CD206 and arginase 1 were markedly decreased compared with the expression in macrophages from PARP-1^−/−^ mice (Fig. 3h, i). These results indicated that PARP-1 promotes an HG-induced phenotype shift in macrophages toward an inflammatory M1 phenotype, and that PARP-1 inhibition improves HG-induced M1/M2 status (Fig. 3h, i).

In addition, the endocytosis of Dil-labeled ox-LDL was abolished in PARP-1^−/−^ peritoneal macrophages compared with WT macrophages (Fig. 3j, k). Together, these results indicated that PARP-1 depletion inhibited the proatherosclerotic phenotype of peritoneal macrophages. Furthermore, we tested whether conditioned medium from macrophages would affect VSMC calcification. Notably, we found that VSMCs treated with conditioned medium from WT macrophages exhibited evident calcification and displayed higher expression levels of Runx2 compared with those in cells treated with medium from PARP-1 depletion macrophages (Fig. 3l, m).

We also observed obvious calcification and decreased M2 differentiation in RAW264.7 cells under osteogenic stimulation especially in HG-osteogenic medium (Supplementary Fig. 2). PJ34 (a PARP inhibitor) attenuated HG-induced macrophage calcification by inhibiting Runx2 expression and enhancing M2 differentiation (Supplementary Fig. 2). We therefore speculated that M1 phenotype switching but not M2 differentiation may worsen atherosclerotic calcification. Together, our results indicated that PARP-1 depletion inhibited osteogenic differentiation by regulating the atherogenic phenotype of macrophages, which in turn may have subsequently decreased diabetic atherosclerotic calcification.

### PARP-1 deficiency attenuates Stat1-mediated diabetic arteriosclerotic calcification

**Fig. 4:**
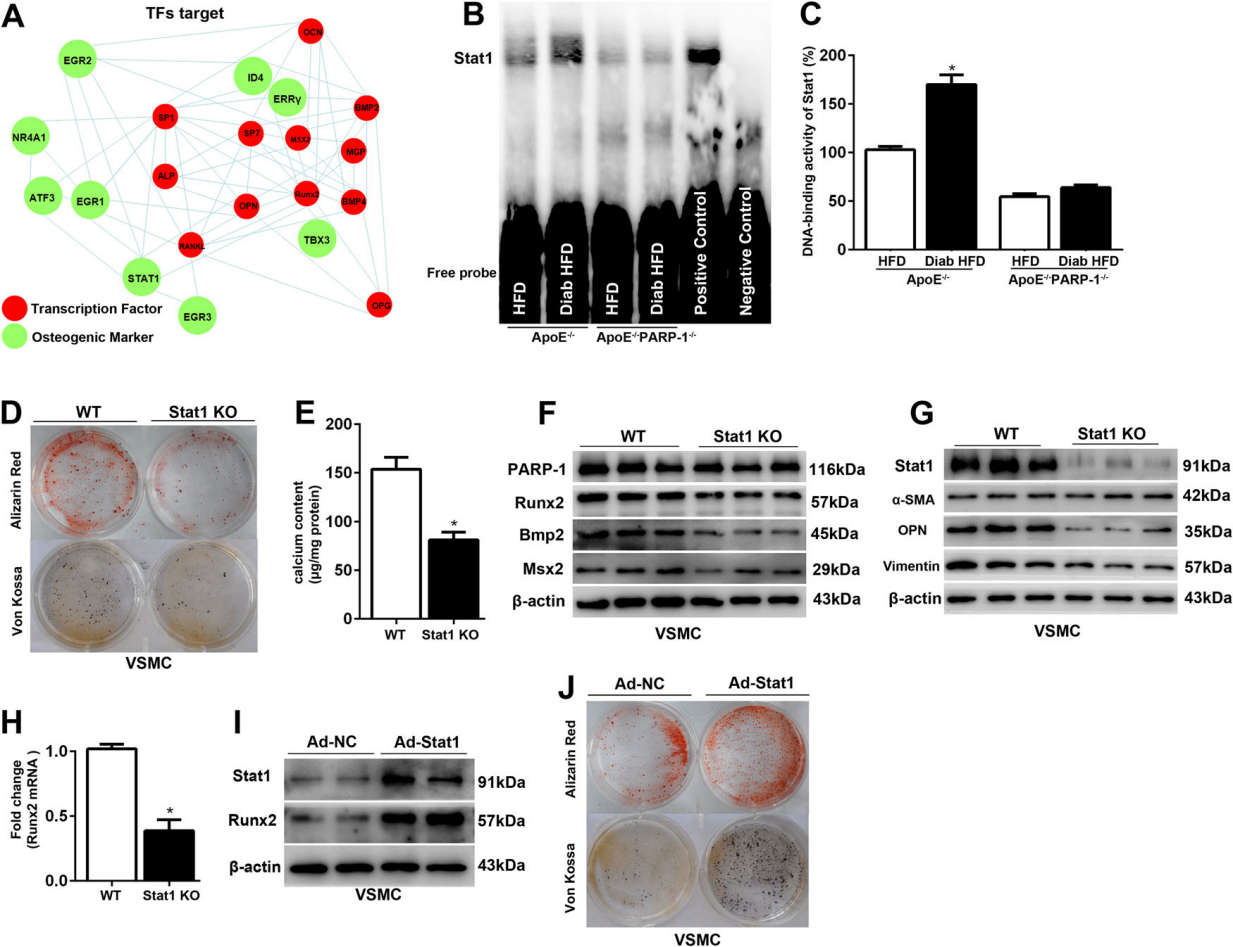
PARP-1 deficiency attenuates Stat1-mediated VSMCs calcification and synthetic phenotype switching. **a** Protein–protein interaction networks illustrate the osteogenic genes downstream of osteogenic TFs. **b**, **c** Stat1 binding activity following PARP-1 deletion in the aortas of diabetic ApoE^−/−^ mice. *n* = 3 in each group. Asterisks indicate statistically significant differences (*P < 0.01, vs. ApoE^−/−^ mice). **d**, **e** Stat1 depletion attenuates VSMC calcification and decreases calcium content after 3-week exposure to osteogenic medium with high glucose (*n* = 5). *P < 0.01, vs. WT VSMC. **f**, **g** Western blot were performed to determine the effect of Stat1 depletion on VSMCs osteogenic differentiation and synthetic phenotype switching (*n* = 5). **h** Real-time PCR analysis were performed to determine the effect of Stat1 depletion on the mRNA expression of Runx2 (*n* = 5). *P < 0.01, vs. WT VSMCs. **i**, **j** Effect of Stat1 overexpression on Runx2 expression and VSMC calcification as measured by Alizarin Red and Von Kossa staining after 2-week exposure to osteogenic medium with high glucose (*n* = 5). The statistical tests are justified as appropriate and meet the assumptions of the tests. The variance between the groups is similar.

To better understand the mechanism by which PARP-1 deletion attenuated diabetic atherosclerotic calcification, protein–protein interaction networks highlighted osteogenic genes downstream of transcription factors (TFs) which were associated with osteogenic differentiation (Fig. 4a). As Stat1 was reported to be an attenuator of Runx2 in osteoblast differentiation^24^, we further found an enhancement of Stat1 transcriptional activity and its reversal by PARP-1 deletion in the aortas of diabetic ApoE^−/−^ mice (Fig. 4b, c). We next ascertained whether Stat1 contributed to diabetic atherosclerotic calcification. Stat1 depletion contributed to the decreased VSMC calcification and calcium content (81.30 ± 8.12 vs. 153.82 ± 12.20, *p* < 0.01) that occurred in osteogenic medium with HG (Fig. 4d, e). Western blot analysis demonstrated that Stat1 depletion impeded the osteogenic gene expression of Runx2, Bmp2, and Msx2 (Fig. 4f). We also confirmed a similar role of Stat1 in HG-induced VSMC synthetic phenotypic transition (Fig. 4g). Notably, overexpression of Stat1 enhanced the expression of Runx2 and thus aggravated calcification in VSMCs (Fig. 4h–j).

**Fig. 5:**
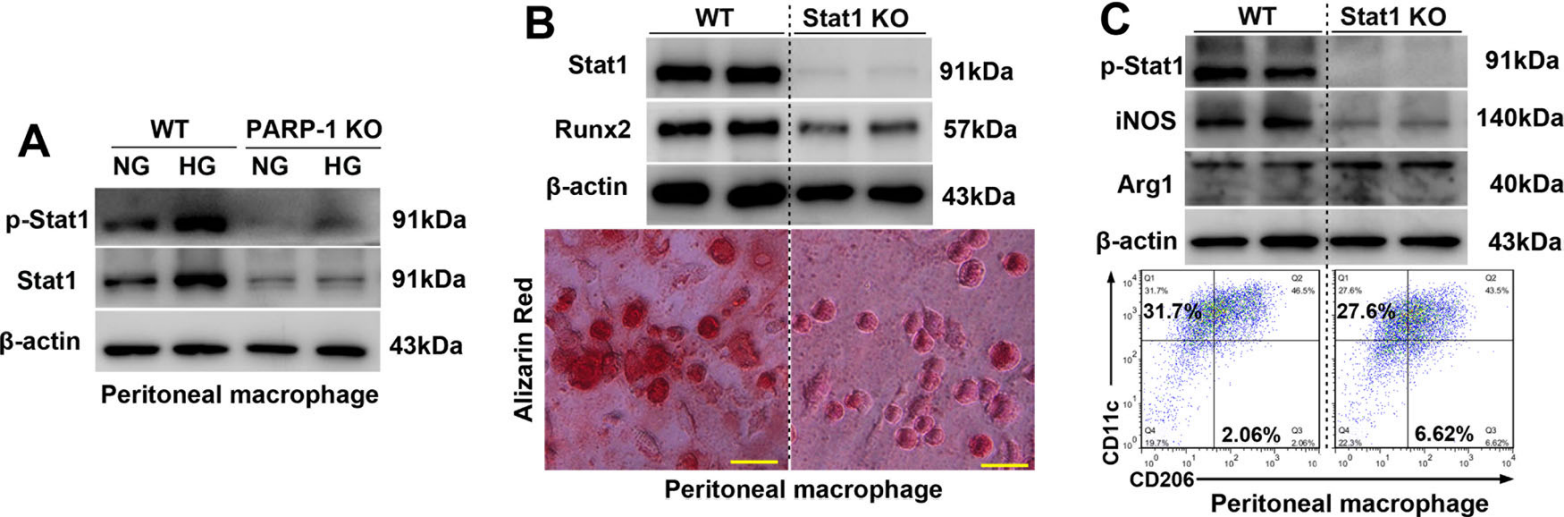
PARP-1 deficiency attenuates Stat1-mediated macrophage calcification and macrophage polarization. **a** Immunoblotting was performed to detect the effect of PARP-1 depletion on total and phosphorylated Stat1 levels (*n* = 5). **b** Stat1 depletion attenuates high glucose-promoted macrophage calcification in vitro (*n* = 5). Scale bar = 10 µm. **c** Immunoblotting and flow cytometry were performed to detect the effect of Stat1 depletion on macrophage phenotype switching (*n* = 5).

To evaluate whether PARP-1 plays a role in the atherogenic phenotype of macrophages by promoting Stat1 phosphorylation, we tested phosphorylated Stat1 (p-Stat1) and total Stat1 expression in cultured peritoneal macrophages. Simultaneously, both phosphorylated Stat1 and total Stat1 increased markedly in WT peritoneal macrophages under HG treatment, although no significant change in PARP-1 deficient macrophages was observed (Fig. 5a). We further determined whether Stat1 depletion could reduce macrophage calcification. Peritoneal macrophages were isolated from Stat1^f/f^ and Stat1^f/f^/lyzM^Cre^ mice. As with PARP-1, Stat1 deletion attenuated HG-induced macrophage calcification by inhibiting Runx2 (Fig. 5b). Furthermore, immunoblotting and flow cytometry indicated that Stat1 depletion also inhibited M1 macrophage phenotype switching (Fig. 5c).

### Stat1 directly binds to the Runx2 promoter and contributes to PARP-1-mediated diabetic arteriosclerotic calcification

**Fig. 6:**
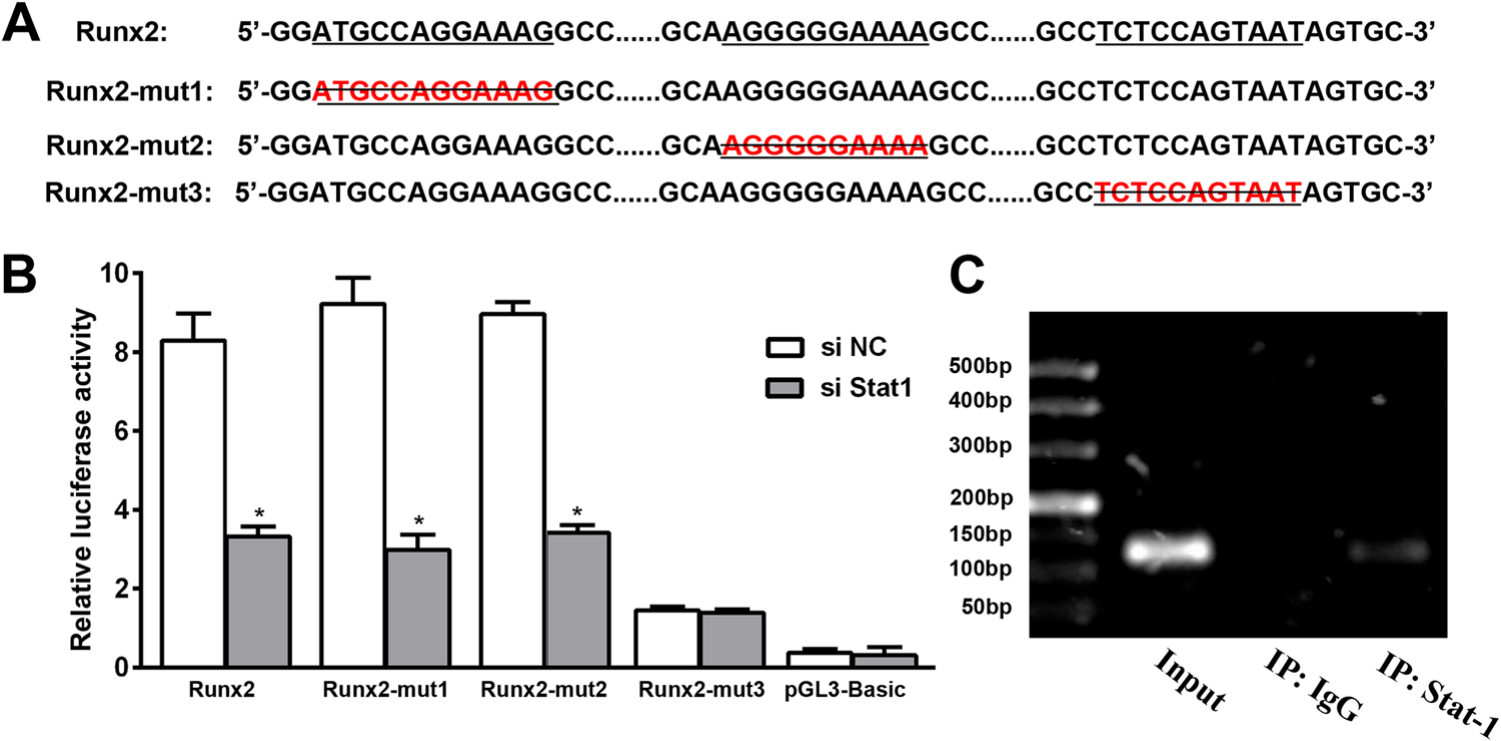
Stat1 directly binds to the Runx2 promoter and contributes to PARP-1-mediated arteriosclerotic calcification. **a** Predicted Stat1 binding site (underlined) within the human *Runx2* promoter. Mutants with deletion of the predicted binding site (Runx2-mut1, Runx2-mut2, and Runx2-mut3) are shown. **b** Luciferase activity assay was performed after transfection with the human *Runx2* promoter or *Runx2* promoter mutants in 293T cells (*n* = 5).*P *<* 0.01, vs. transfection with con siRNA. **c** ChIP assay to verify binding of Stat1 to the *Runx2* promoter (*n* = 5). The statistical tests are justified as appropriate and meet the assumptions of the tests. The variance between the groups is similar.

Considering the Stat1-dependent regulation of both the mRNA and protein expression of Runx2, we hypothesized that Stat1 may regulate Runx2 through transcriptional activation. We searched for potential Stat1 binding sites within the mouse and human *RUNX2* promoter using PROMO and JASPAR databases. There were no mouse Stat1 information in JASPAR database, but we identified three potential Stat1 recognition motifs (5′-ATGCCAGGAAAG-3′, 204 bp upstream, 5′-AGGGGGAAAA-3′, 144 bp upstream, and 5′-TCTCCAGTAAT-3′, 67 bp upstream) of the human *RUNX2* transcription start site (Fig. 6a). To confirm that the predicted site of the *RUNX2* promoter is required for transcriptional activity, we constructed intact promoter-reporter plasmids containing the predicted *RUNX2* promoter region and mutations of the predicted binding site. Human embryonic kidney 293T cells were simultaneously transfected with an intact or mutant *RUNX2* promoter-reporter plasmid along with control siRNA or Stat1 siRNA. As depicted in Fig. 6b, a luciferase assay was used to demonstrate that the −67 bp promoter region is required for *RUNX2* transcriptional activity. Furthermore, a significant reduction of *RUNX2* promoter luciferase activity was observed following treatment with Stat1 siRNA, implying that Stat1 regulates Runx2 through transcriptional activation. We next performed a quantitative ChIP assay to verify binding of Stat1 to the *RUNX2* promoter using specific primers covering −67 to −57 bp of the *RUNX2* promoter region. As expected, Stat1 specifically bound to the −67 to −57 bp site of the *RUNX2* promoter (Fig. 6c). We further found Stat1 overexpression upregulated osteogenic genes including Runx2, Bmp2, and Msx2 in HA-VSMCs (Supplementary Fig. 3).

**Fig. 7:**
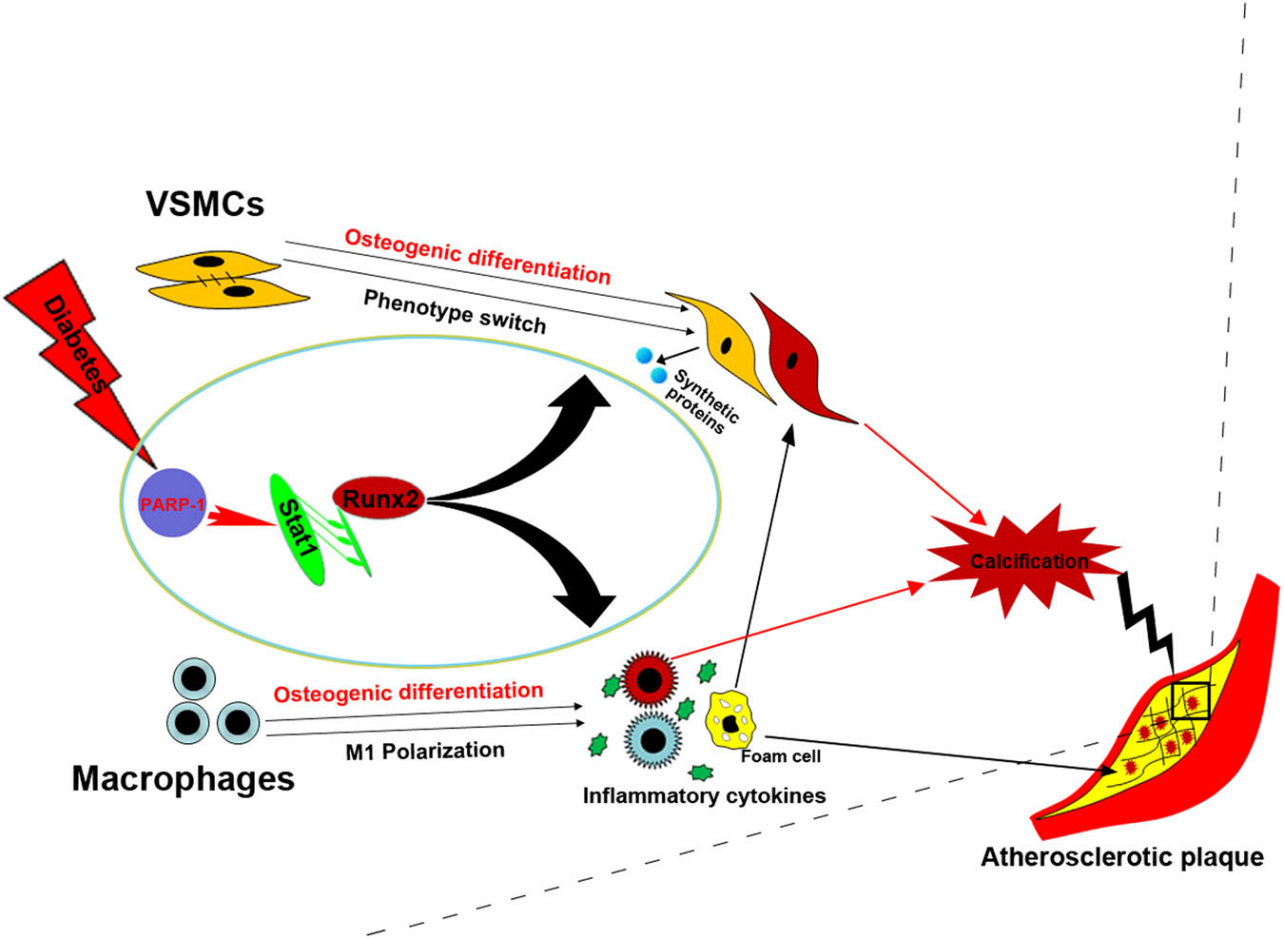
Proposed working schematic of PARP-1 depletion-mediated attenuation of atherosclerotic calcification in diabetes. Diabetes activates Runx2 expression and induces the osteogenic differentiation of both VSMCs and macrophages. Concurrently, diabetes promotes phenotype switching of VSMCs from the contractile phenotype to a dedifferentiated synthetic phenotype, and of macrophages to a proinflammatory M1 phenotype, which in turn aggravates VSMC calcification. PARP-1 acts on Stat1 transcription, which functions as a regulator of Runx2 expression and osteogenic differentiation. PARP-1 depletion reversed the hyperglycemia-induced synthetic phenotype switching of VSMCs and macrophage polarization by targeting Stat1. As a result, PARP-1 depletion suppresses diabetic atherosclerotic calcification.

We proposed working schematic of PARP-1 depletion-mediated attenuation of atherosclerotic calcification in diabetes (Fig. 7). Diabetes drives Runx2 expression and induces the osteogenic differentiation of both VSMCs and macrophages. Concurrently, diabetes promotes phenotype switching of VSMCs from the contractile phenotype to a dedifferentiated synthetic phenotype, and of macrophages to a proinflammatory M1 phenotype, which in turn aggravates VSMC calcification. In addition, PARP-1 acts on Stat1 transcription, which directly binds the Runx2 promoter and regulates osteogenic differentiation. PARP-1 depletion reversed the hyperglycemia-induced synthetic phenotype switching of VSMCs and macrophage polarization by targeting Stat1. As a result, PARP-1 depletion suppresses diabetic atherosclerotic calcification.

## Discussion

We demonstrate for the first time that PARP-1 plays a novel role in the regulation of osteogenic differentiation in both VSMCs and macrophages regardless of the HG milieu. Genetic deletion of PARP-1 downregulated osteogenic marker genes in atherosclerotic plaques and isolated VSMCs as well as in macrophages. More importantly, these findings were more obvious under HG milieus. Mechanistically, our results suggest that PARP-1 regulates hyperglycemia-accelerated arteriosclerotic calcification by targeting Stat1/Runx2 axis.

In previous studies, male ApoE^−/−^ background mice were challenged with a HFD for 24 weeks^6^ or 30 weeks^25^ to generate atherosclerotic calcification in vivo. Our data reveal that HFD for 12 weeks is sufficient to induce obvious arteriosclerotic calcification in diabetic male ApoE^−/−^ mice. Therefore, we mainly study the mechanism that hyperglycemia accelerate atherosclerotic calcification. We found that PARP-1 deletion attenuated atherosclerotic calcification, reduced the content of aortic calcium, and decreased vessel stiffening in diabetes. Ingenuity Pathway Analysis (IPA) indicated TFs including STAT1, ATF3, EGR1, EGR2, EGR3, ID4, NR4A1, and TBX3 acted on osteogenic genes and may therefore subsequently contribute to atherosclerotic calcification in vivo. Among these TFs, we focused on Stat1, a known attenuator of Runx2 in osteoblast differentiation^24^. Meanwhile, PARP-1 deletion inhibited the transcriptional activity of Stat1, another therapeutic target for osteogenic differentiation. Similarly, we observed that Stat1 depletion in VSMCs attenuated calcification. A previous study has reported that Stat1 negatively regulates Runx2 expression and thus contributes to osteoblast differentiation^24^. However, our results showed an opposite role of Stat1 with respect to mineralization in VSMCs and osteoblasts. Specifically, Stat1 overexpression upregulated Runx2 expression and exacerbated osteogenic differentiation in VSMCs, whereas Stat1 inhibition decreased calcium content and calcification. Our results were consistent with the recent report by Smyth et al.^26^ that a gain-of-function mutation in the Stat1 gene lead to aortic calcification in a patient. Interestingly, we further identified a potential Stat1 recognition motif (5′-TCTCCAGTAAT-3′) for Stat1-mediated activation of the *RUNX2* promoter using PROMO and JASPAR databases. Luciferase activity and ChIP assay results confirmed the binding of Stat1 to the *RUNX2* promoter.

Previous studies indicated that VSMC phenotype switching with concomitant reduction of contractile proteins (α-SMA, SM-22α) and increased synthetic proteins (OPN, MGP) aggravated plaque instability^27,28^. In addition, VSMC phenotypic transition was associated with vascular calcification^23^. We further illustrated the effect of PARP-1 deletion on VSMC phenotypes. We found that HG aggravated phenotype switching in osteogenic medium, promoting VSMC conversion from a contractile phenotype to a dedifferentiated synthetic phenotype. As expected, PARP-1 deletion reversed the phenotype switching of VSMCs. Studies have also shown that HG stimulated OPN expression and induced the alteration of VSMC phenotype in vivo and in vitro^4^. Our results further suggest that PARP-1 deletion increased VSMC markers and decreased the expression of synthetic phenotype markers in VSMCs cultured in osteogenic medium by targeting Stat1, which may in turn contribute to arteriosclerotic calcification and plaque stability. These data indicate that the PARP-1/Stat1/Runx2 axis in VSMCs plays an important role in diabetic atherosclerotic calcification.

To date, the precise vascular cell type participating in arteriosclerotic calcification has remained undefined and the contribution of macrophages to atherosclerotic calcification is controversial. To elucidate the function of macrophages in atherosclerotic calcification in vivo and in vitro, we cultured macrophages in osteogenic medium for 3 weeks and generated macrophage-specific PARP-1 deletion mice on an ApoE^−/−^ background. We observed evident calcification in both RAW264.7 and peritoneal macrophages after 3-week exposure to osteogenic medium with HG treatment. In addition, colocalization of TRAP and CD68 revealed that macrophages independently participated atherosclerotic calcification in vivo. This was consistent with the study of Byon et al.^29^, which indicated that macrophage infiltration was associated with calcified atherosclerotic lesions. Moreover, a genetic fate mapping study revealed that VSMCs and bone marrow derived cells accounted for ~80% and 20% of Runx2-positive cells in calcified atherosclerotic vessels of ApoE^−/−^ mice, respectively^30^. These studies demonstrated the independent contribution of macrophages to atherosclerotic calcification^7,30–32^. Alternatively, other studies have suggested that macrophages could enhance VSMC calcification by releasing proinflammatory cytokines in an in vitro coculture model^33^. Sun et al.^6^ reported that osteogenic VSMCs promoted macrophage infiltration into the calcified lesion. Notably, despite the interplay between VSMCs and macrophages, our study provides definitive evidence that the osteogenic differentiation of macrophages directly promotes the pathogenesis of atherosclerotic calcification in vivo and in vitro. In accordance with this observation, macrophage-specific PARP-1 depletion inhibited arteriosclerotic calcification in diabetic ApoE^−/−^ mice. These data indicate that the PARP-1/Stat1/Runx2 axis in macrophages also plays an important role in atherosclerotic calcification.

Notably, macrophages displayed an enhanced M1 phenotype in parallel with obvious calcification in osteogenic medium containing HG, which implied that the proinflammatory M1 phenotype drove macrophage calcification. Macrophages tended to express higher levels of iNOS, TNF-α, and IL-12, and to switch to the M1 phenotype under HG conditions^34^. The study of Torres-Castro et al.^35^ also showed enhanced expression of CD11c and iNOS, as well as downregulation of Arginase1 following exposure of macrophages to HG. In the current study, we found that HG aggravated the phenotype switching of macrophages from an anti-inflammatory M2 phenotype to a proinflammatory M1 phenotype. PARP-1 or Stat1 deletion reversed the HG-promoted macrophage calcification and Runx2 expression in peritoneal macrophages. Simultaneously, PARP-1 depletion enhanced M2 differentiation and inhibited the M1 differentiation by targeting Stat1. Thus, we speculate that M1 phenotype switching may worsen macrophage calcification. This is in agreement with the findings of Fu et al.^7^, which indicated that the shift in macrophage phenotype toward a proatherosclerotic state drove atherosclerotic calcification. In addition, we found that PARP-1 deletion abolished the endocytosis of Dil-labeled ox-LDL in peritoneal macrophages. Furthermore, previous studies have reported that Stat1 deficiency instituted by using a bone marrow transplantation technique could not only suppress M1 polarization but also decrease foam cell formation in macrophages and reduce atherosclerosis in ApoE^−/−^ mice^36–38^. Collectively, these results indicate that PARP-1 depletion inhibited the osteogenic and atherogenic phenotype of macrophages by targeting Stat1 and may subsequently decrease atherosclerotic calcification in diabetes. Finally, enhanced M2 differentiation owing to PARP-1 deletion may also contribute to plaque stability^39^. Together, these results suggest that PARP-1 deletion in macrophages contributes to atherosclerotic calcification by inhibiting the osteogenic and atherogenic phenotype of macrophages.

In conclusion, the findings of the present study have uncovered a common mechanism by which diabetes accelerates atherosclerotic calcification. Our results indicate that PARP-1 depletion in VSMCs or macrophages significantly inhibits osteogenic differentiation in vitro and in vivo. These novel findings identify PARP-1 inhibition as a potential therapeutic target for preventing atherosclerotic calcification.

## Materials and methods

### Animal models

PARP-1^−/−^ mice were obtained from the Jackson Laboratory. PARP-1^f/f^ mice and Stat1^f/f^ mice were generated by View Solid Biotechnology Inc. under the combined use of CRISPR/Cas9 and Cre/LoxP. SMA^Cre^ and lyzM^Cre^ transgenic mice were kindly provided by Dr. Wen-cheng Zhang. ApoE^−/−^PARP-1^−/−^ were generated by crossing PARP-1^−/−^ mice with ApoE^−/−^ mice. VSMC- or macrophage-specific PARP-1 deletion mice on an ApoE^−/−^ background were generated by crossing floxed mice with SMA^Cre^ or lyzM^Cre^ mice, and then crossbreeding with ApoE^−/−^ mice to generate ApoE^−/−^/PARP-1^f/f^/SMA^Cre^, ApoE^−/−^/PARP-1^f/f^/lyzM^Cre^.

### Animal experiments

We fed 12-week-old male ApoE^−/−^ background mice high fat western diet (HFD) for 12 weeks. The mice were rendered diabetic by daily intraperitoneal injections of STZ (Sigma, St. Louis, MO) at a dose of 50 mg/kg for 5 consecutive days. To determine the treatment of PARP inhibitor PJ34 (Sigma, St. Louis, MO) on diabetic arteriosclerotic calcification, diabetic ApoE^−/−^ mice on HFD were injected intraperitoneally with PJ34 at a dose of 10 mg/kg every other day for 12 weeks. To determine the role of PARP-1 and to what extent hyperglycemia contributes to arteriosclerotic calcification in vivo, ApoE^−/−^; ApoE^−/−^PARP-1^−/−^; diabetic ApoE^−/−^; and diabetic ApoE^−/−^PARP-1^−/−^ animals were randomly grouped. To determine the role of VSMC-specific, macrophage-specific PARP-1 in diabetic atherosclerotic calcification, mice were grouped as follows: diabetic ApoE^−/−^/PARP-1^f/f^; ApoE^−/−^/PARP-1^f/f^/SMA^Cre^; ApoE^−/−^/PARP-1^f/f^/lyzM^Cre^. To ensure at least five mice in each group could be used to statistical analysis, we chose eight mice into each group. If the mouse died during the study, it would be excluded. All experimental protocols conformed to the Guide for the Care and Use of Laboratory Animals published by the US National Institutes of Health and were approved by the animal ethics committee of Shandong University. The investigator was not blinded during the study. After 12 weeks, mice were anesthetized with sodium pentobarbitone and their organs were immediately harvested.

### Aortic calcification assay

Calcium mineral deposits in aortic sections were detected by Alizarin Red and Von Kossa staining (Sigma, St. Louis, MO) as described previously^6^. The percentage of positively stained area for each aortic section was quantified using ImageJ software (NIH). Calcium content in the aortic tissues was measured as previously described^6^. Dissected aortas were briefly incubated at 70 °C for 30 min to obtain their dry weight and decalcified with 0.6 mM HCl at 37 °C for 48 h. The vascular calcium concentration was quantified using the Calcium Colorimetric Assay Kit (Sigma, St. Louis, MO) and normalized by the dry weight of the aortas, which was expressed as fold-change compared with control.

### Measurement of PWV

PWV was measured via a modification of the transit time method using echocardiography with Vevo770 imaging system (Visual Sonics) as previously detailed^40^. Mice were anesthetized with continuous inhalation of 1–2% isoflurane. The curvilinear distance from the aortic arch to the abdominal aorta was measured (D2 − D1; in mm) by the exact coordinates of the Doppler sample volumes. The time delay (T2 − T1; in ms) was measured relative to the simultaneously recorded electrocardiogram signal. PWV was calculated by the formula (D2 − D1/T2 − T1), expressed as m/s.

### Immunohistochemistry and immunofluorescence assays

Frozen sections were incubated with primary antibodies [rabbit anti-PARP-1 (Santa Cruz, Cat#SC-7150) and anti-Runx2 (Abcam, Cat# ab192256)] overnight at 4 °C. Sections were further washed and exposed to a secondary antibody at 37 °C for 30 min. The antibody binding signals were visualized with diaminobenzidine.

For fluorescence imaging, after incubation with antibodies (mouse anti-Runx2 1:100 (Abcam, Cat#ab76956), rabbit anti-TRAP 1:100 (Abcam, Cat#ab191406), mouse anti-CD68 1:200 (Abcam, Cat#ab201340), rabbit anti-vimentin 1:200 (Cell Signaling Technology, Cat# 5741), or mouse anti-α-SMA 1:200 (Abcam, Cat# ab7817) overnight at 4 °C, sections were washed followed by incubation with the appropriate secondary antibodies (Alexa Fluor 488 and 549 goat anti-rabbit; Alexa Fluor 549 and 488 anti-mouse 1:200) for 30 min. 4′, 6-diamidino-2-phenylindole (DAPI, Invitrogen) was used for nuclei staining. Specific fluorescence imaging was performed by laser-scanning confocal microscopy (LSM710, Carl Zeiss).

### Ingenuity pathway analysis and protein–protein interaction networks

IPA and protein–protein interaction networks were performed by Genechem Co., Ltd.

### Cell culture

Aortic primary VSMCs were isolated from mouse aortas as described previously^6^. All experiments were performed with VSMCs at passages 3–5. Peritoneal macrophages were isolated from thioglycollate-injected mice as previously described^7^. Briefly, mice were intraperitoneally injected with 1 ml of 3.0% thioglycollate. After 72 h, the cells were perfused from the peritoneal cavity and then cultured in Dulbecco’s modified Eagle medium (Gibco, Grand Island, NY). RAW264.7 were purchased from the American Type Culture Collection (ATCC, Manassas, VA).

### In vitro calcification

Calcification was induced by culturing the cells in osteogenic medium containing 10 mM β-glycerophosphate for 3 weeks with or without HG (27.5 mM). Medium was changed every 2–3 days. Alizarin Red and Von Kossa staining were conducted to detect calcification in separate culture dishes. Total calcium content in the cell lysates was quantified using a Calcium Colorimetric Assay Kit (Sigma, St. Louis, MO).

### SiRNA transfection

To effect Stat1 inhibition, siRNA (listed in Table S3) or control at 50 nM were incubated with Lipofectamine RNAiMAX Transfection Reagent (Invitrogen, NY, USA) in Opti-MEM (Gibco, Grand Island, NY) per manufacturer instruction. Recombinant adenovirus constructs carrying Stat1 cDNA were purchased from Vigene Bioscience (Jinan, China). Adenovirus encoding murine WT Stat1 (Ad-Stat1) or control virus (Ad-NC) was transduced into VSMCs at a multiplicity of infection of 10. After 24 h, virus-containing medium was replaced with fresh complete medium.

### Real time reverse transcription (RT)-PCR

Total RNA was extracted using TRIzol reagent (Invitrogen, NY, USA) and reverse-transcribed into cDNA. SYBR Green-based RT-PCR was performed using a sequence detection system (IQ5 Real-Time PCR cycler, Bio-Rad Laboratories). Relative expression was normalized to that of β-actin. Primer details for real-time quantification are shown in Table S3.

### Western blotting assay

Briefly, equal amounts of the proteins from cell lysates were separated by 10% sodium dodecyl sulfate-polyacrylamide gel electrophoresis and transferred to polyvinylidene difluoride membranes and then incubated with specific antibodies at 4 °C overnight. Horseradish peroxidase-conjugated secondary antibodies were added and incubated at room temperature for 1 h. The bound primary antibodies were detected by chemiluminescence (Millipore, Billerica, MA). Bands were quantified by ImageJ software. Expression levels were normalized to the control. The following primary antibodies were employed: anti-PARP-1 (1:500, Santa Cruz, Cat#SC-7150), anti-Runx2 (1:1000, Cell Signaling Technology, Cat#12556), anti-Bmp2 (1:1000, Abcam, Cat#ab14933), anti-Msx2 (1:1000, Sigma, Cat#HPA005652),anti-OPN (1:1000, Abcam, Cat#ab8448), anti-Stat1 (1:1000, Cell Signaling Technology, Cat#14994), anti-p-Stat1 (Tyr701, 1:1000, Cell Signaling Technology, Cat#5375), anti-vimentin (1:1000, Cell Signaling Technology, Cat# 5741), anti-α-SMA (1:1000, Abcam, Cat#ab32575), anti-iNOS (1:1000, Abcam, Cat#ab178945), anti-Arg1 (1:1000, Cell Signaling Technology, Cat#93668), and anti-β-actin (1:5000, Sigma, Cat#SAB2100037).

### Luciferase activity assay

293T cells were transiently cotransfected with a Runx2 promoter luciferase reporter plasmid (pGL3-Runx2-Luc) and a Renilla reporter plasmid (pRL-TK) using Lipofectamine 3000 transfection reagent (Invitrogen). Luciferase activity was determined using the Dual Luciferase Assay Kit (Promega, WI, USA).

### Chromatin immunoprecipitation assay (ChIP)

ChIP assay was performed using a ChIP Assay kit (Millipore). VSMCs were fixed with 4% formaldehyde and sonicated on ice 10 times using a Vibra-Cell ultrasonic processor (Sonics) for 15 s with a 60 s interval between each sonication. For immunoprecipitation, anti-Stat1 antibody, or normal IgG were used. DNA fragments in the final immunoprecipitates were determined by PCR amplification. Primers used for this analysis are listed in Table S3.

### Electrophoretic mobility shift assay (EMSA)

To detect DNA-protein binding, EMSA was performed using the Light shift chemiluminescence EMSA kit (Thermo Fisher Scientific, Rockford, USA) according to manufacturer protocol. Nuclear extracts were prepared from aortas using the NE-PER Nuclear and Cytoplasmic Extract kit (Pierce, USA). The labeled oligonucleotide probe designed to encompass putative PARP-1 binding sites in the *Stat1* promoter was: 5′-CATGTTATGCATATTCCTGTAAGTG-3′.

### Measurement of Dil-ox-LDL uptake

Peritoneal macrophages were plated in dishes and incubated with DiI-ox-LDL for 4 h. Then, fluorescence imaging was performed by laser-scanning confocal microscopy (LSM710, Carl Zeiss).

### Flow cytometric analysis

After exposure to osteogenic medium, Peritoneal macrophages or RAW264.7 macrophages were colabeled with PE anti- CD11c and APC anti-CD206 antibodies (eBioscience, CA, USA). Then the cells were examined using a FACS Calibur system (BD Biosciences, San Jose, CA, USA) and data analyzed using Flow Jo software.

### Statistical analysis

Data are presented as the means ± standard deviation. Statistical tests were performed using SPSS16.0 software and GraphPad Prism software version 6.0. A two-tailed unpaired *t* test was used for statistical comparisons of two groups, and one-way ANOVA was used for multiple groups.

## Supplementary information

Supplementary Figure legends

Supplement Fig1

Supplement Fig2

Supplement Fig3

Supplemental Table-1

Supplemental Table-2

Supplemental Table-3
