# Correction: Loss of PARP-1 attenuates diabetic arteriosclerotic calcification via Stat1/Runx2 axis

**DOI:** 10.1038/s41419-020-2292-8

**Published:** 2020-02-06

**Authors:** Peng Li, Ying Wang, Xue Liu, Bin Liu, Zhao-yang Wang, Fei Xie, Wen Qiao, Er-shun Liang, Qing-hua Lu, Ming-xiang Zhang

**Affiliations:** 1 Department of Pharmacology, College of Pharmacy, Xinxiang Medical University, Xinxiang, China; 2 The Key Laboratory of Cardiovascular Remodeling and Function Research, Chinese Ministry of Education, Chinese Ministry of Health and Chinese Academy of Medical Sciences, Department of Cardiology, Qilu Hospital of Shandong University, Jinan, China; 3 Department of Cardiology, The Second Hospital of Shandong University, Jinan, Shandong China

**Keywords:** Differentiation, Calcification

**Correction to**: **Cell Death and Disease**

10.1038/s41419-019-2215-8 published online 10 January 2010

Since publication the authors have noticed an error in the corresponding author information. The corresponding author information should be as follows: Correspondence: Er-shun Liang (liangershun@126.com) or Qing-hua Lu (lqhzhy@163.com) or Ming-xiang Zhang (zhangmingxiang@sdu.edu.cn).

This has been corrected in the PDF and HTML versions.

The authors would like to apologise for any inconvenience caused.

