# Supplementary material for: Loss of PARP-1 attenuates diabetic arteriosclerotic calcification via Stat1/Runx2 axis
Source: Cell Death Dis. 2020 Jan 10;11(1):22. doi: 10.1038/s41419-019-2215-8 (PMC6954221; doi:10.1038/s41419-019-2215-8)
Supplement: Supplementary file 1 — Supplementary Figure legends [file 41419_2019_2215_MOESM1_ESM.docx]

Supplementary Figure legends

Supplementary Fig.1. PARP-1 inhibition attenuates diabetic atherosclerotic calcification and decreases vessel stiffening.

(A and B) PARP inhibitor PJ34 treatment decreased Alizarin Red positively stained atherosclerotic lesion area of the aortic roots.

(C and D) PJ34 treatment decreased aortic calcium content and Pulse wave velocity.

Scale bar = 100 µm. Bar values represent the means ± SD. n = 8 in each group. Asterisks indicate statistically significant differences (**P* < 0.01, vs. ApoE^−/−^ HFD mice). The statistical tests are justified as appropriate and meet the assumptions of the tests. The variance between the groups is similar.

Supplementary Fig.2. PARP inhibitor PJ34 attenuates diabetic atherosclerotic calcification in vitro.

VSMCs or Raw264.7 macrophages were exposed to osteogenic medium with high glucose for 3 weeks. Calcification was measured by Alizarin Red staining. (A and B) PJ34 treatment (10 μM) attenuated VSMC calcification and decreased TRAP-positive cells by inhibiting Runx2 expression. (C, D and E) PJ34 increased M2 macrophage markers and attenuated high glucose-promoted macrophage calcification by inhibiting Runx2 activity. Scale bar = 100 µm.

Supplementary Fig.3. Effect of Stat1 overexpression on expression of osteogenic genes including Runx2, Bmp2 and Msx2 in human aortic (HA)-VSMC.
