# Supplementary figures and images for: Loss of PARP-1 attenuates diabetic arteriosclerotic calcification via Stat1/Runx2 axis
Source: Cell Death Dis. 2020 Jan 10;11(1):22. doi: 10.1038/s41419-019-2215-8 (PMC6954221; doi:10.1038/s41419-019-2215-8)

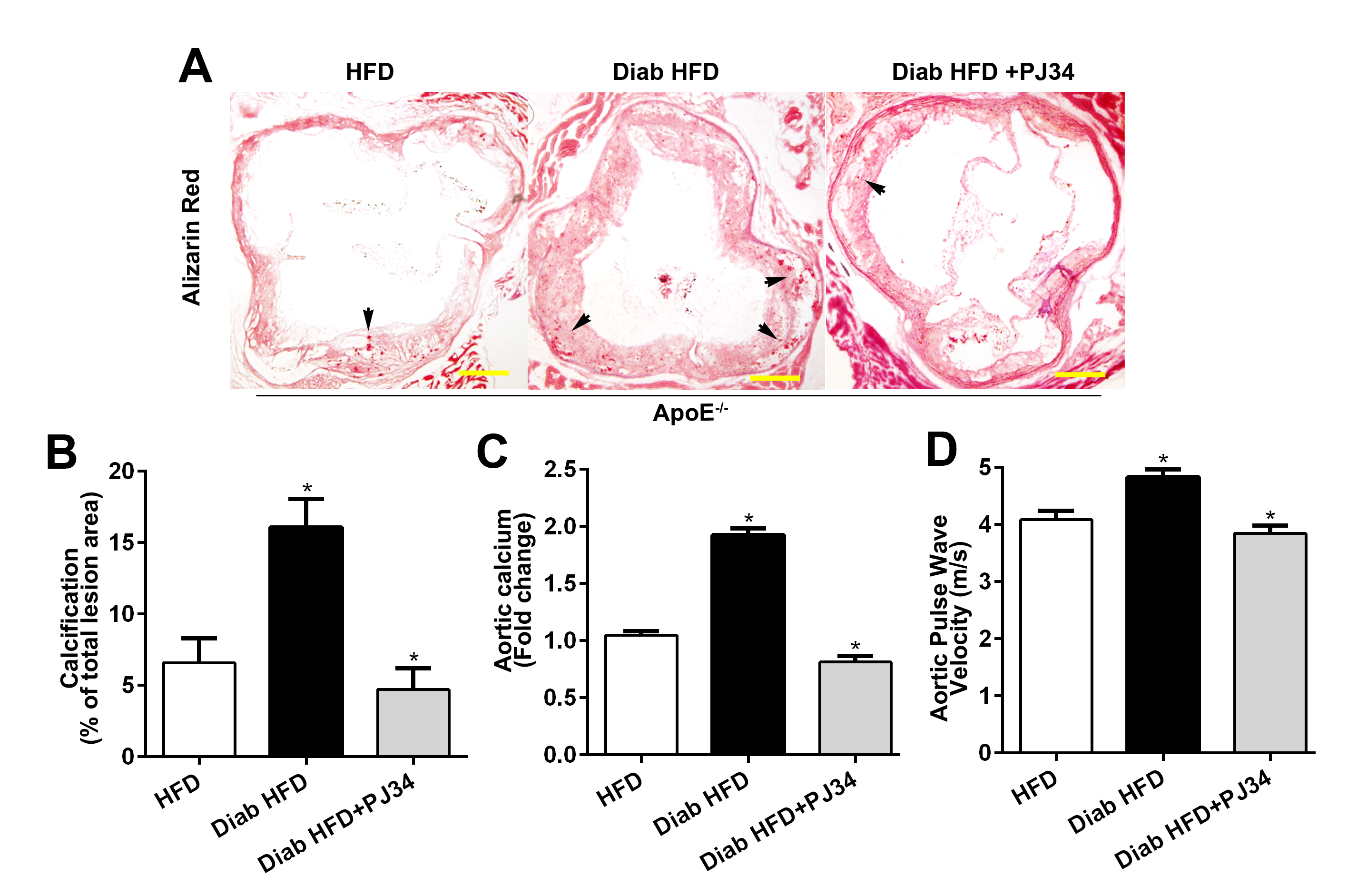

Supplement: Supplementary file 2 — Supplement Fig1 [file 41419_2019_2215_MOESM2_ESM.tif]

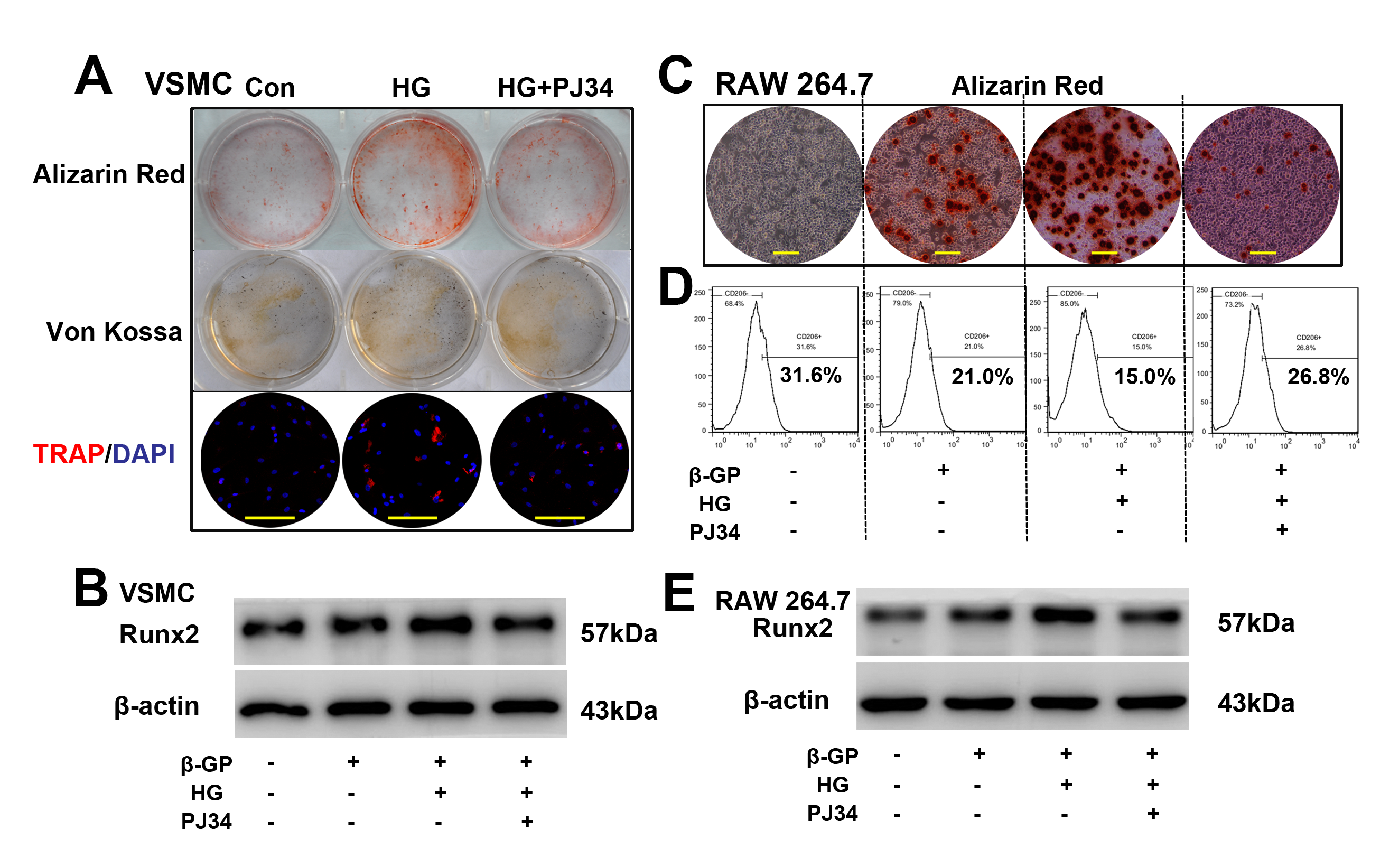

Supplement: Supplementary file 3 — Supplement Fig2 [file 41419_2019_2215_MOESM3_ESM.tif]

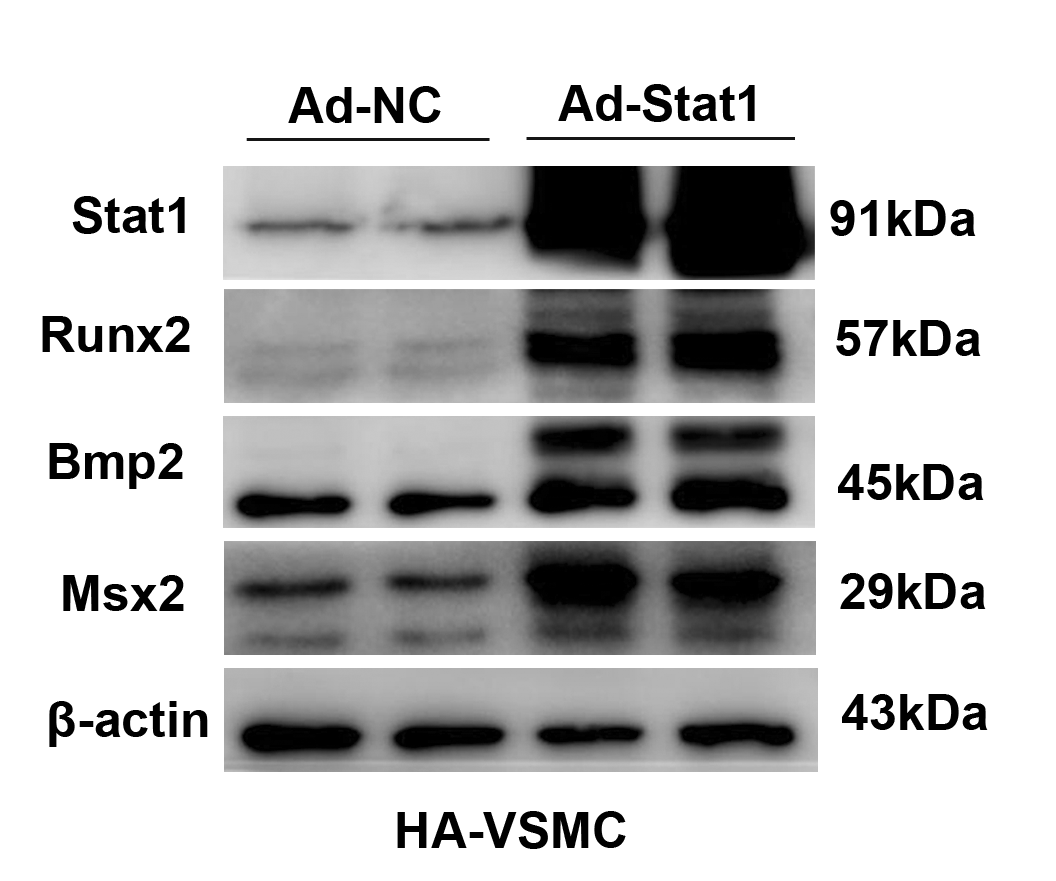

Supplement: Supplementary file 4 — Supplement Fig3 [file 41419_2019_2215_MOESM4_ESM.tif]
