# Supplementary material for: Loss of PARP-1 attenuates diabetic arteriosclerotic calcification via Stat1/Runx2 axis
Source: Cell Death Dis. 2020 Jan 10;11(1):22. doi: 10.1038/s41419-019-2215-8 (PMC6954221; doi:10.1038/s41419-019-2215-8)
Supplement: Supplementary file 5 — Supplemental Table-1 [file 41419_2019_2215_MOESM5_ESM.doc]

**Table S1.** General Metabolic Parameters of PARP-1 Deletion Mice on an ApoE^−/−^ Background Treated With High-Fat Western Diet for 12 Weeks

|  | ApoE^−/−^ | Diab ApoE^−/−^ | PARP-1^−/−^ApoE^−/−^ | Diab PARP-1^−/−^ApoE^−/−^ |
| --- | --- | --- | --- | --- |
| Body weight (g) | 31.10 ± 0.59 | 28.56 ± 0.88*^#^ | 31.13 ± 0.79 | 28.76 ± 0.71*^#^ |
| Blood glucose (mmol/l) | 7.95 ± 0.75 | 20.20 ± 1.33*^#^ | 7.85 ± 0.69 | 19.46 ± 1.17*^#^ |
| Total cholesterol (mmol/l) | 26.46 ± 0.69 | 34.34 ± 0.68*^#^ | 26.16 ± 0.78 | 33.39 ± 2.38*^#^ |
| Triglyceride (mmol/l) | 1.60 ± 0.05 | 1.95 ± 0.07*^#^ | 1.58 ± 0.07 | 1.91 ± 0.08*^#^ |
| Calcium (mmol/l) | 2.39 ± 0.04 | 2.30 ± 0.08 | 2.37 ± 0.02 | 2.37 ± 0.03 |
| Phosphorus (mmol/l) | 3.42 ± 0.22 | 3.15 ± 0.18 | 3.25 ± 0.14 | 3.41± 0.14 |

n=8 in each group. Data are expressed as mean ± SEM.

**P*<0.01 vs. ApoE^−/−^; ^#^*P*<0.05 vs. PARP-1^−/−^ApoE^−/−^.
