# Supplementary material for: Loss of PARP-1 attenuates diabetic arteriosclerotic calcification via Stat1/Runx2 axis
Source: Cell Death Dis. 2020 Jan 10;11(1):22. doi: 10.1038/s41419-019-2215-8 (PMC6954221; doi:10.1038/s41419-019-2215-8)
Supplement: Supplementary file 6 — Supplemental Table-2 [file 41419_2019_2215_MOESM6_ESM.doc]

**Table S2.** General Metabolic Parameters of Diabetic PARP-1^f/f^ , PARP-1^f/f^SMA^Cre^, and PARP-1^f/f^lyzM^Cre^ Mice on an ApoE^−/−^ Background Treated with High-Fat Western Diet for 12 Weeks

|  | PARP-1^f/f^ ApoE^-/-^ | PARP-1^f/f^ SMA^Cre^ApoE^−/−^ | PARP-1^f/f^ lyzM^Cre^ApoE^−/−^ |
| --- | --- | --- | --- |
| Body weight (g) | 28.86 ± 1.28 | 28.85 ± 1.02 | 27.89 ± 0.93 |
| Blood glucose (mmol/l) | 19.34 ± 1.11 | 19.83 ± 1.50 | 18.88 ± 1.02 |
| Total cholesterol (mmol/l) | 33.44 ± 2.25 | 33.66 ± 1.27 | 31.81 ± 2.30 |
| Triglyceride (mmol/l) | 1.91 ± 0.11 | 1.96 ± 0.08 | 1.93 ± 0.05 |

n = 8 in each group. Data are expressed as the means ± SEM.
