# Supplementary material for: Loss of PARP-1 attenuates diabetic arteriosclerotic calcification via Stat1/Runx2 axis
Source: Cell Death Dis. 2020 Jan 10;11(1):22. doi: 10.1038/s41419-019-2215-8 (PMC6954221; doi:10.1038/s41419-019-2215-8)
Supplement: Supplementary file 7 — Supplemental Table-3 [file 41419_2019_2215_MOESM7_ESM.doc]

**Table S3.** siRNA sequences and primer sequences for RT-PCR and ChIP

|  | Forward primer (5′-3′) | Reverse primer (5′-3′) |
| --- | --- | --- |
| si Stat1 Homo | GCGUAAUCUUCAGGAUAAUTT | AUUAUCCUGAAGAUUACGCTT |
| *Runx2* Mus | AGGGACTATGGCGTCAAACA | GGCTCACGTCGCTCATCTT |
| *β-actin* Mus | CAAAGACCTGTACGCCAACAC | TCATAGTCCGCCTAGAAG |
| ChIP: Homo *Runx2* | CTTAACCTTACAGGAGTTTGGGCT | CTTTCTCTCACAAAAGGCTTGTGG |
